# Optofluidic Tunable Lenses for In-Plane Light Manipulation

**DOI:** 10.3390/mi9030097

**Published:** 2018-02-26

**Authors:** Qingming Chen, Tenghao Li, Zhaohui Li, Jinlin Long, Xuming Zhang

**Affiliations:** 1 Department of Applied Physics, The Hong Kong Polytechnic University, Hong Kong 999077, China; qing-ming.chen@connect.polyu.hk (Q.C.); 14900295r@connect.polyu.hk (T.L.); 2 School of Electronics and Information Technology, Sun Yat-Sen University, Guangzhou 510275, China; li_zhaohui@hotmail.com; 3 School of Chemistry and Chemical Engineering, Fuzhou University, Fuzhou 350116, China; jllong@fzu.edu.cn; 4 Shenzhen Research Institute of the Hong Kong Polytechnic University, Shenzhen 518057, China

**Keywords:** optofluidics, microfluidics, in-plane liquid lens, lab-on-a-chip

## Abstract

Optofluidics incorporates optics and microfluidics together to construct novel devices for microsystems, providing flexible reconfigurability and high compatibility. Among many novel devices, a prominent one is the in-plane optofluidic lens. It manipulates the light in the plane of the substrate, upon which the liquid sample is held. Benefiting from the compatibility, the in-plane optofluidic lenses can be incorporated into a single chip without complicated manual alignment and promises high integration density. In term of the tunability, the in-plane liquid lenses can be either tuned by adjusting the fluidic interface using numerous microfluidic techniques, or by modulating the refractive index of the liquid using temperature, electric field and concentration. In this paper, the in-plane liquid lenses will be reviewed in the aspects of operation mechanisms and recent development. In addition, their applications in lab-on-a-chip systems are also discussed.

## 1. Introduction

Nowadays, miniaturized systems are playing important roles in both academic research and industrial applications. More elements and functions have been incorporated into a single chip, reducing the cost and improving the performance at the same time. Optofluidics combines optics and microfluidics together to construct novel elements for lab-on-a-chip applications [1,2,3,4,5,6,7]. By replacing the solid materials with liquids, it enables the flexible modulation of optical properties and improves compatibilities. Compared with its solid counterpart, optofluidics has some unique merits [8,9,10,11], such as flexible tunability, good compatibility, small size and easy fabrication, etc. It has been intensively studied by numerous research communities [1,2,3,7,12,13]. A number of applications have been achieved using the optofluidic techniques, such as chemical and biological detection [4,5,14], particle manipulation [15,16], optofluidic laser [13,17], tunable waveguides [18,19], and reconfigurable optofluidic lens [12]. Among them, the optofluidic lens is the most extensive exploited part. In conventional optical systems, the optical lens is used to change the propagation of light and reshape the beam. The solid lens has constant refractive index (RI) and a fixed focal length. The optical modulation is achieved by mechanical movement, which is complicated and poorly scalable. While the optofluidic lens enables easy modulation of the optical properties by either changing the lens geometry or tuning RI of the liquid.

By the manipulation of liquids in microscale, reconfigurable optofluidic lens has been demonstrated in a small chip. According to the propagation of the beam, the liquid lenses can be generally classified into two categories: out-of-plane lens and in-plane lens [12]. The former manipulates the beam in the direction perpendicular to the substrate of microfluidic chip [20], which is similar to the conventional lens. It can be used to replace the solid lens in some miniature optical systems that require variable focusing. The latter deals with the light in the direction parallel to the substrate of microfluidic chip, providing an effective way to manipulate the beam in a microfluidic network. Therefore, the sample and the probe beam can be delivered in the same liquid layer, enhancing the interaction of light and matter. Thus, it promises better scalability and more complexity for optofluidic networks. Some reviews have reported the development and applications of the optofluidic lenses [12,21,22,23]. Nam-Trung Nguyen gave a comprehensive review of the optofluidic lenses on their categories and working principles [12]. It focused on the schematic designs of different optofluidic lenses and some characteristic parameters, such as the response time and the tunability of focal length and RI. It also listed some liquids that are widely used in optofluidics. Mishra et al. reported the general characteristics and characterization methods of the optofluidic lenses, including the actuation methods and the spherical aberration [22]. In particular, Mishra had deeply discussed the aberration control, which is very important in optical imaging. Xu et al. discussed the development of dielectrophoretically tunable optofluidic lenses [23]. And Krogmann presented the design, fabrication and optical properties of the electrowetting based micro-optical components [24]. However, a comprehensive description of in-plane liquid lenses and their perspectives in optofluidic networks is still required. This review focuses on the in-plane optofluidic lenses, including the working mechanisms and their applications in lab-on-a-chip systems.

**Figure 1:**
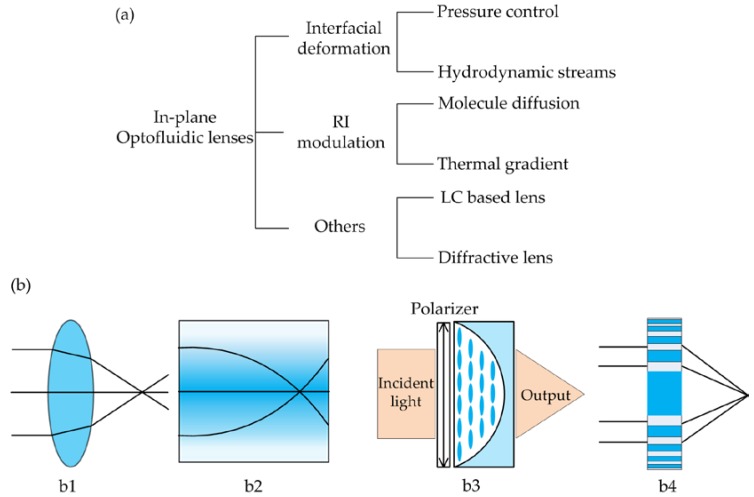
In-plane optofluidic lenses categories and the working principles: (**a**) The in-plane liquid lenses are classified into three types of lenses according to their working principles; (**b**) schematic diagrams of the in-plane liquid lens: (**b1**) is the interfacial deformation lens; (**b2**) is RI modulation (gradient index) lens; (**b3**) is the liquid-crystalbased lens (LC: the blue ellipses) and (**b4**) is the diffractive lens (i.e., the Fresnel zone plate).

Figure 1a summarizes the categories of the in-plane lenses and Figure 1b explains the corresponding working principles. Most of the reported designs of in-plane optofluidic lenses can classified into two types: refractive lens and gradient index (GRIN) lens. The former often makes use of interfacial deformation and the latter of RI modulation. The rest of reported designs can be generally grouped into the others in this review. In the refractive lens, the beam is refracted at the smooth fluidic interface of immiscible liquids (see Figure 1(b1)). The focal length is usually tuned by changing the lens geometry. In a microfluidic chip, there are numerous ways to modify the curvature of the fluidic interface, for example, pressure control [25], hydrodynamic modulation [26,27], electrowetting [24] and dielectrophoresis [23]. Among them, the pressure control and the hydrodynamic modulation are more popular in regulating in-plane lenses. In the GRIN lens, solution diffusion [28] or thermal diffusion [29,30] can be used to establish a RI gradient profile, in which the rays are bent gradually and then focused to a point (Figure 1(b2)). A large RI gradient (usually over 0.1) is achievable in a microscale region, resulting in tight focusing and wide tunable range. The refractive lens and the GRIN lens are often independent of the polarization and the wavelength of the incident light due to the use of isotropic and low-dispersion liquids (e.g., water, ethanol, ethylene glycol). In addition, there are some other lenses that aim at polarization separation or wavelength selection, such as birefringent liquid lens [31] and diffractive optofluidic lens [32]. For example, liquid crystal (LC) has been used to construct a polarization-dependent liquid lens [31]. Figure 1(b3) displays the schematic of the LC liquid lens, in which the LC molecules can be realigned by applying a sufficiently strong electrical field. As a result, incident beams of different polarization directions would experience different RIs and have different focal lengths. Another kind of lens is Fresnel zone plate (FZP), which is based on the diffraction rather than refraction. As shown in Figure 1(b4), an optofluidic FZP can be achieved for light manipulation by filling liquid into the periodic microstructure. Different parts of the diffracted lights interference constructively with each other to form a focal point. The FZP depends on the incident wavelength, providing another freedom of tunability.

This article has five sections. The first gives an introduction of optofluidic lenses. In the second, the in-plane optofluidic lenses will be discussed according to their operation mechanisms. Then, some applications of the in-plane liquid lenses will be presented in the third section. There is a brief discussion in section four. The last part is the conclusion.

## 2. Classification of In-Plane Optofluidic Lenses

In this part, the in-plane optofluidic lenses will be reported based on their operation mechanisms. Firstly, some example of the refractive lenses based on the fluidic interfaces will be presented. Then the GRIN lenses are discussed. In addition, the in-plane liquid lenses based on other methods are also discussed at the end of this section.

### 2.1. Interfacial Deformation

**Figure 2:**
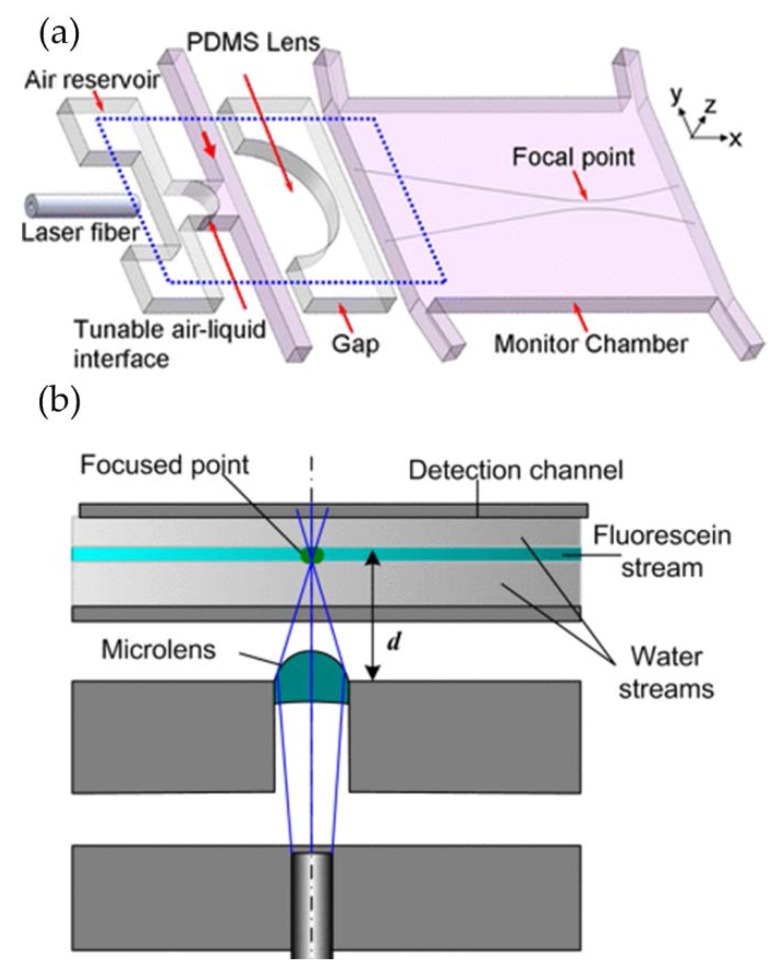
Pressure-control liquid lenses: (**a**) liquid-air interface tuned by flow rate control [25]; (**b**) pneumatically droplet tunable lens [34].

The most straightforward method to construct an in-plane optofluidic tunable lens is to use the interfaces between immiscible streams (or liquid-air interface), where the interfacial curvature can be modified by numerous microfluidic techniques [25,26,33]. The general working principle is depicted in Figure 1(b1). In the case of in-plane lens, the geometry modulation can be achieved either by the pressure control or by the hydrodynamic streams. In the pressure-control liquid lens, the curvature of the liquid-air interface is modified by external pumping [25,34]. Tony Huang’s group proposed a reconfigurable in-plane liquid lens using fluidic pressure to tune the liquid/air interface in a microfluidic chip [25]. As shown in Figure 2a, this microlens consists of a reconfigurable divergent liquid-air interface and a static polydimethylsiloxane (PDMS) lens. The liquid flows through a straight channel and traps the air in the chamber, forming a liquid-air interface. By adjusting the flow rate, it changes the pressure inside the channel as well as the interfacial radius. It demonstrated the continuous modulation of the focal length by tuning the flow velocity. Behind the lens, there is a chamber for experimental raytracing. Another pressure-controllable liquid-air in-plane lens is demonstrated by Dong et al. [34]. By precisely locating a liquid droplet at the T-shape junction, a tunable in-plane liquid lens is formed in the microchannel (see Figure 2b). This microlens has a tunable focal length from a few hundreds of micrometers to infinite. It can be pneumatically repositioned and removed inside the predefined microchannel. The geometry of the chamber can also be used as a tunable lens by modifying the shape using the pressure control [35]. The pressure-control liquid-air interface is governed by the Laplace law:

(1) $$\Delta P=2\gamma \kappa =\gamma \left(\frac{1}{R_{1}}+\frac{1}{R_{2}}\right)$$

where *γ* is the surface tension coefficient between the liquid and air, *κ* is the mean curvature of the liquid-air interface. *R*_1_ (in horizontal) and *R*_2_ (in vertical) are the principal curvature radii of the interface. The ideal liquid-air interface is spherical in both horizontal and vertical directions. The external pump is used to balance at the pressure drop at the interface [25,34].

**Figure 3:**
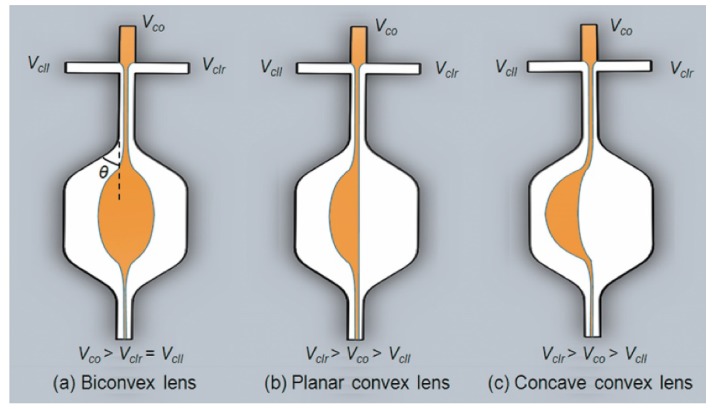
Different curvatures of tunable liquid microlenses via the control of laminar flow rate [36]: (**a**) biconvex lens; (**b**) plano-convex lens; (**c**) concave-convex lens.

Another way to change the geometry of the in-plane liquid lens is to use the hydrodynamic modulation [26,27,33], in which the fluidic curve is formed and controlled by hydrodynamic force. In this case, two or more immiscible streams (the liquid core and the liquid cladding) are pumped into a specific microchannel to form reconfigurable interfaces. Tuning the ratio of the flow streams enables to continuously modulate the fluidic interfaces. It should be noted that the optical properties of the hydrodynamic stream liquid lens are dependent on the shape of the fluidic chamber. Seow et al. demonstrated a tunable liquid lens by injecting three flow streams into a rectangle-shaped expansion chamber [36], where the liquid with a higher RI acts as the core and the other two streams with a lower RI act as the cladding. Figure 3 shows the schematic designs of the liquid lenses, *V_co_* is the flow rate of the core, *V_cll_* and *V_clr_* are the flow rates of the left and right claddings, respectively. A biconvex lens is formed when *V_co_* > *V_clr_* = *V_cll_*, see Figure 3a. And the curvature radius becomes smaller with a higher cladding flow rate. By increasing the value of *V_clr_*, the microlens becomes plano-convex (Figure 3b) and then concave-convex (Figure 3c), respectively. Both collimation and focusing have been demonstrated in this type of microlens by tuning the flow rates.

**Figure 4:**
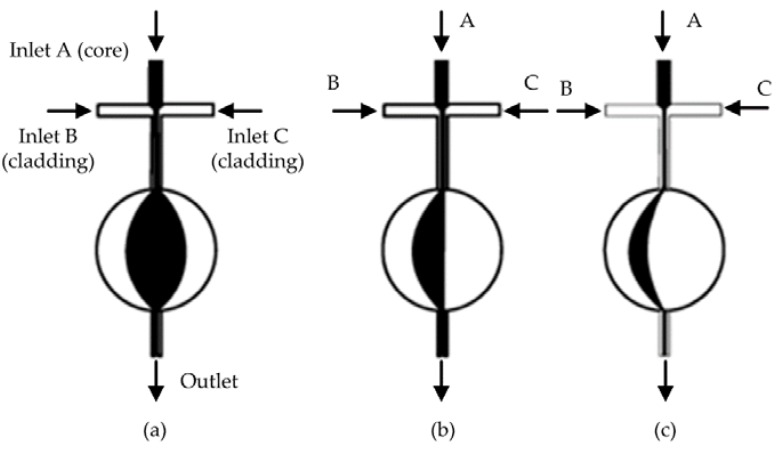
Reconfigurable optofluidic lenses with a circular lens chamber [37]: the lens shape is modified by adjusting the flow rates of the core and cladding streams. (**a**) biconvex lens; (**b**) plano-convex lens; (**c**) concave-convex lens.

**Figure 5:**
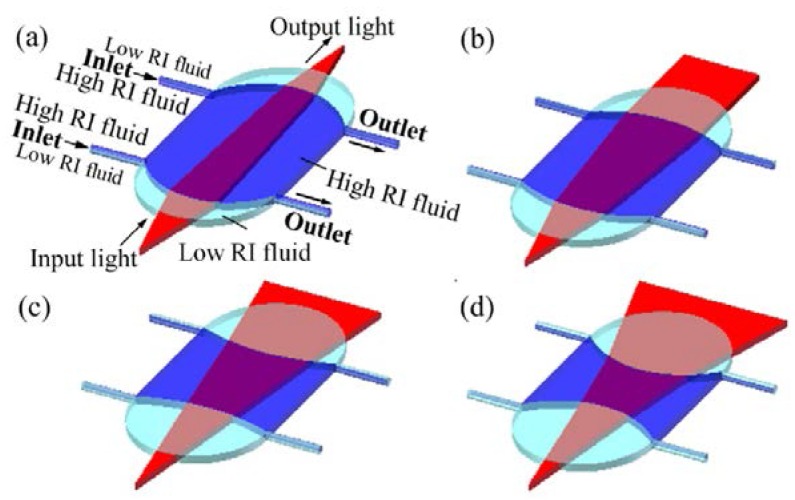
Hydrodynamically reconfigurable optofluidic lens, in which the liquid core (in blue) is sandwiched by the liquid claddings [27]. (**a**) The liquids form a biconvex lens and the beam is focused. (**b**) The beam is collimated when the interface curvature becomes smaller. (**c**,**d**) A biconcave lens is obtained and the beam becomes divergent.

**Figure 6:**
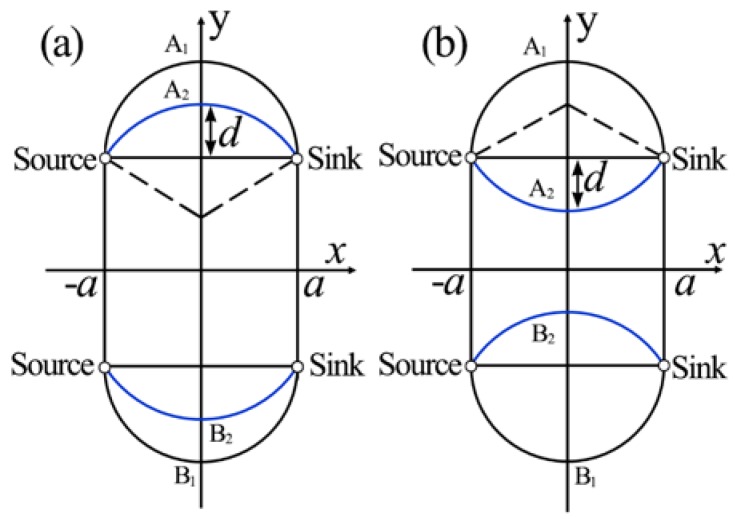
The coordinate of microlens model [27].

Another design of liquid lens uses the circular chamber as shown in Figure 4. Song et al. reported the modeling and experimental results of a tunable lens by injecting three laminar streams into a circular chamber [37]. The liquid-core liquid-cladding lens with perfect curvatures was formed by the circular design. Figure 4 describes the schematic design of the circular liquid lens. In the symmetric state, the lens has a biconvex shape as shown in Figure 4a. By further increasing the flow rate of inlet C, it can tune the lens into the plano-convex shape (Figure 4b) and then the concave-convex shape (Figure 4c). The curvature radius can be tuned from that of the chamber to infinity. As the width of the channel is much smaller than that of the expansion chamber, the model can be approximately described as a source-sink pair model [37]. A reconfigurable biconcave lens was demonstrated by Li et al. [38]. They used the combination of pressure driven flow and electro-osmosis to realize both focusing and diverging in a rectangle chip. Fang et al. proposed a hydrodynamically reconfigurable optofluidic lens, which can be tuned from biconcave to biconvex [27]. Figure 5 depicts the operation principle of the liquid lens. The chamber with two convex ends is used to realize the modulation from biconcave to biconvex. Two immiscible liquids with different RIs are injected into the expansion chamber, where the liquid core (with higher RI) is sandwiched by the liquid claddings (with lower RI). The curvature of the interface is modified by tuning the flow ratio of the core and cladding streams. When the cladding flow is low, the liquid core expands outside into the cladding area, resulting in a biconvex lens (see Figure 5a,b). By increasing the rate of the cladding flow, the curvature of the liquid lens decreases. With the further increase of the cladding flow, the liquid core is compressed into a biconcave shape and the lens becomes negative, as shown in Figure 5c,d. The modulation from biconvex (positive) to biconcave (negative) lens has been demonstrated by adjusting the flow rate. They proposed a two-dimensional quadrupolar flow model to analyze the operation of the liquid lens [27]. As shown in Figure 6, the model has two sources at the left side and two sinks at the right side. The sources and sinks were regarded as dimensionless points. By combining the flow model and the theory of thick lens together, an equation was derived to describe the focal length:

(2) $$f=\frac{-n_{1}r^{2}}{2\left(n_{1}-n_{0}\right)\left[\left(n_{1}-n_{0}\right)\left(s+b\right)-n_{1}r\right]}$$

where *n*_0_ and *n*_1_ are the RIs of the liquid cladding and liquid core, respectively. And *b* is half of the distance between the two sources, *r* is the curvature radius of the liquid interface. The parameter *s* equals to *d* or -*d* when the interface is positive or negative, respectively. By using the combination of a tunable biconvex lens and a reconfigurable liquid prism. Chao et al. demonstrated the controlling of the focal length and the deviation angle of the beam [39].

### 2.2. Refractive Index (RI) Modulation

As mentioned above, the RI modulation is another way to alter the optical properties of fluidic components. A simple method to change the RI of the liquid medium is to replace one with another. Seow et al. proposed a tunable planar optofluidic lens using a PDMS lens chamber [40]. By filling the chamber with the mixer of two miscible liquids, the RI was tuned from 1.33 to 1.63. The RI of medium is dependent on several physical properties such as concentration [28,41] and temperature [30]. It can be also changed by external electric field, acoustic field and mechanical strain. The optical propertis of the optofluidic device can be tuned through the modulation of the RI profile, which is also very popular in solid optics. For instance, in a graded index optical fiber, rays follow sinusoidal paths and cross each other periodically. Similarly, rays bend gradually and focus to a focal point, forming the GRIN lens. Compared with the solid materials, the RI modulation of liquid is much easier. By simply changing the concentration of the solutions, the RI change over 0.1 can be achieved [21]. A variety of optofluidic waveguides [19,42,43] and lenses [28,44] base on the diffusion of two miscible solutions have been demonstrated. Temperature gradient is another effective way to form a RI gradient in fluid [30].

**Figure 7:**
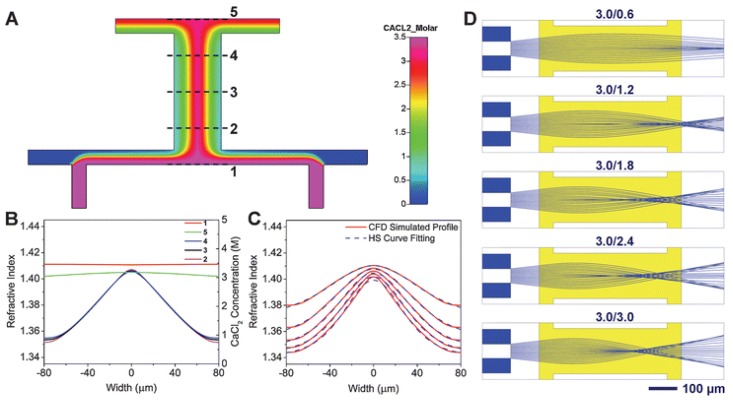
L-GRTN lens with two degrees of freedom [28]. (**A**) Simulated refractive index profile and ray tracing. (**B**) Cross-sectional refractive index distribution at different locations along the flow direction (1, 2, 3, 4 and 5 as indicated in a). (**C**) Refractive index distribution along line 3 (defined in a) at different flow rates. (**D**) Ray tracing results in different flow conditions (3.0/0.6 represents CaCl_2_ flow rates = 3.0 µL m^−1^ and H_2_O flow rate = 0.6 µL m^−1^, respectively).

A simple method to form a RI gradient within liquid medium is solute diffusion. In a laminar flow inside the microchannel, the concentration gradient is determined by the solution diffusion [45], which can be modulated by the flow rate control. Therefore, a graded RI profile can be achieved using the solution diffusion. Yang et al. proposed an optofluidic RI gradient for lightwave bending and manipulation through the diffusion between ethylene glycol and deionized water [19], in which the RI can be tuned from 1.34 to 1.42. Mao et al. demonstrated a reconfigurable liquid gradient index (L-GRIN) lens with two degrees of freedom using CaCl_2_ solution as the core and deionized water as the cladding [28]. As shown Figure 7A, the two liquids (the CaCl_2_ solution and DI water) are injected into the microfluidic chip to establish the gradient profile by diffusion of laminar flows. The rays bend gradually when they meet the RI gradient. Tuning the flow rates of the liquids enables not only to change the focal length, but also to shift the focused beam away from the optical axis, providing another freedom for adaptive optics. Figure 7B depicts the RI distribution along lines 1–5. The RI profile inside the channel follows a hyperbolic secant (HS) function as

(3) $$n^{2}\left(x\right)=n_{s}^{2}+\left(n_{0}^{2}-n_{s}^{2}\right){\mathrm{sech}}^{2}\left(\alpha x\right)$$

where *n*(*x*) is the RI at the given position, *n*_0_ is the RI at the center, *n_s_* is the lowest RI in the liquid medium and *α* is the gradient parameter. Changing the flow rate enables the modulation of the RI profile as well as the focal length of the lens. Figure 7C shows the RI along line 3 at different flow rates. The ray tracing simulated results in different flow conditions are shown in Figure 7D. It can also shift the focus away from the center using an asymmetric RI profile.

**Figure 8:**
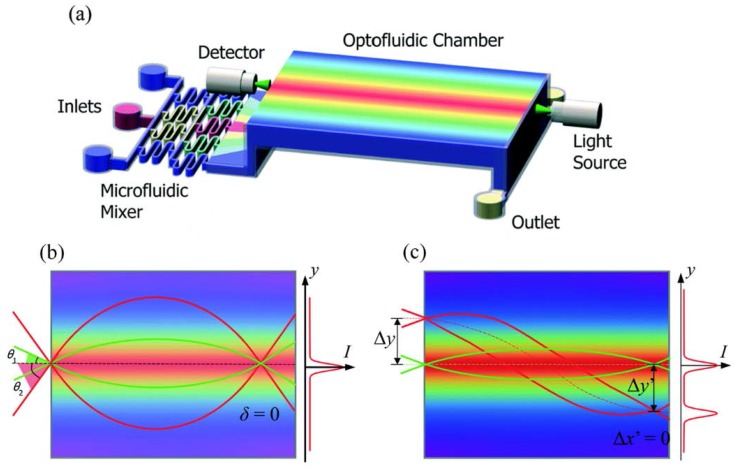
Schematic and working principle of the optofluidic lens [44]. (**a**) Design of the optofluidic chip; (**b**) Spherical aberration; (**c**) Field curvature aberration.

Zhao et al. further improved the performance of the diffusion based optofluidic lens by upgrading the lens design [44], see Figure 8a. By adding a fluidic mixer before the lens section, a HS RI profile can be achieved by precisely controlling the flow rates of the mixer. Borrowed the idea from aberration-free Maxwell’s fisheye lens, such a structure is demonstrated to have a lower spherical aberration (see Figure 8b). It is able to focus the beams to different shifted positions on the same focal plane (Figure 8c).

**Figure 9:**
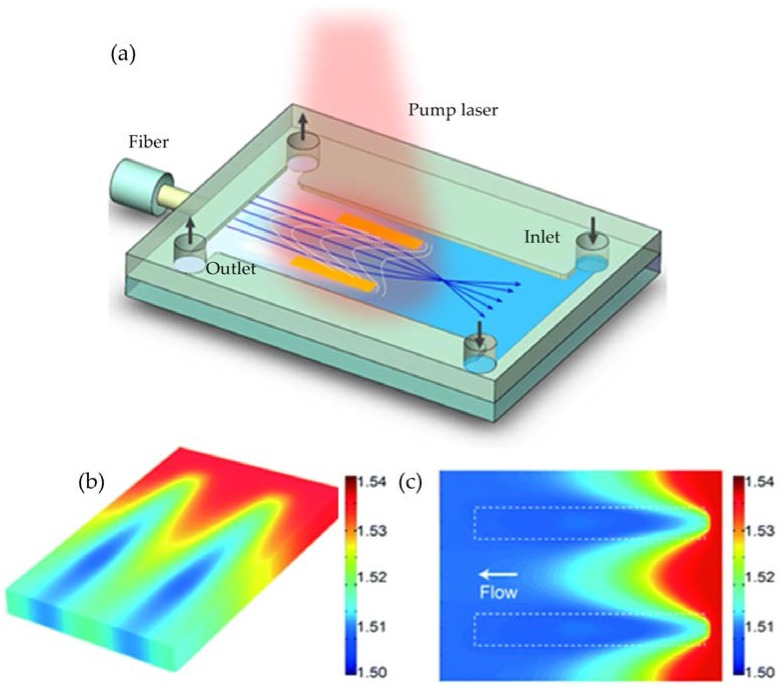
Optofluidic thermal lens using laser-induced thermal gradient [29]: (**a**) schematic design; (**b**) 3D and (**c**) 2D RI profiles.

Temperature conduction is another effective way to form a graded RI profile for beam manipulation in microfluidics. According to the thermal lens effect, the RI decreases linearly while the temperature is increased. Therefore, the RI is lower at the hot region. The rays gradually bend while experiencing an inhomogeneous temperature field. As the magnitude of the thermal conduction coefficient is about two orders larger than that of the molecular diffusion coefficient, the thermal lens effect promises a faster response speed. But the thermos-optics coefficient is relative small, which has a value of 1~10 × 10^−4^ K^−1^. For instance, water has a thermo-optics coefficient of −1.2 × 10^−4^ K^−1^ at 0~80 °C. The thermal-induced RI is at the order of 0.01, which is much smaller as compared to that derived from the concentration gradient. Tang et al. proposed a thermal-induced optical waveguide by the streams at different temperatures [18]. It utilized two streams at higher temperature (the cladding) to sandwich another stream at lower temperature (the core) to form a temperature gradient across the channel. By simply changing the flow rate, the optical properties of the liquid waveguide can be modified. In our previous work, we presented an optofluidic tunable lens using the laser-induced thermal gradient, in which a RI gradient is established in microscale for focusing. As shown in Figure 9, a pump laser is utilized to illuminate the two metal patterns (the yellow pads in Figure 9a), which absorb the light and heat up the flowing liquid (benzyl alcohol, d*n*/d*T* = 4 × 10^−4^ K^−1^). A temperature induced RI gradient is established inside the microchannel for beam manipulation. Different from the conventional GRIN lenses, this laser-induced thermal lens has a 2D RI gradient, in which the cross-sectional RI follows the square-low parabolic function as described by

(4) $$n\left(r,z\right)=n_{c,z}\sqrt{1-A_{z}r^{2}}$$

where *n*(*r*,*z*) is the RI at point (*r*,*z*) and *z* is the coordinate position along the flow direction. *n_c,z_* is the RI at the central position (*r* = 0,*z*), and *A*_z_ is the parabolic parameter. The simulated 3D- and 2D-RI profiles are shown in Figure 9b,c, respectively. The rays bend gradually and focus to a point while passing the gradient section in between the two metal strips (see Figure 9a). This optofluidic lens allows to use only one liquid. The pump laser enables noncontact modulation and free relocation of the lens region.

### 2.3. Others

Apart from the above mentioned liquid lenses, there are other types of in-plane optofuidic lenses that can also be used for beam manipulation in microfluidic networks.

In the conventional hydrodynamic liquid-liquid lens, isotropic liquids are used as the core and the cladding, which are polarization independent. However, a polarization-dependent device may find special applications in which the polarized light is preferred. A commonly used polarizable liquid is the LC that has a state between isotropic liquid and totally anisotropic solid crystal, resulting in a partially anisotropic fluid. One of the LC phases is nematic, which is usually uniaxial. It has a preferred long axis and a short axis. The nematic LCs have the fluidic properties similar to ordinary organic liquids, but they can be well aligned by a sufficiently strong electric field. Therefore, the nematic LCs have been widely used in electrically reconfigurable optical devices, such as liquid crystal display. Numerous LC lenses have also been demonstrated [46]. The incident light experiences different RIs according to its polarization. The effective RI can be expressed by

(5) $$n_{eff}=n_{o}n_{e}\sqrt{\frac{1}{n_{o}^{2}\cos^{2}\theta +n_{e}^{2}\sin^{2}\theta }}$$

where *θ* is the angle between the LC rod and the polarization of the incident light, *n_e_* and *n_o_* are the exordinary RI and the ordinary RI of the LC molecule, respectively. It is noted that *n_eff_* varies from *n_o_* to *n_e_* according to the wave polarization.

**Figure 10:**
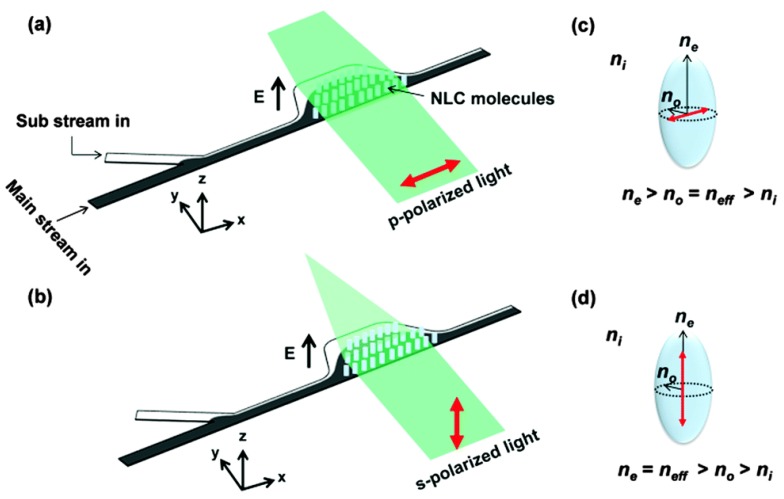
Schematic of the birefringent optofluidic lens [31]: the focusing effect of (**a**) p-polarized and (**b**) s-polarized light. Index ellipsoids are used to describe the RI of (**c**) p-polarized and (**d**) s-polarized light. The red arrows indicate the direction of polarization.

Wee et al. demonstrated an in-plane optofluidic birefringent lens by manipulating the streams of a nematic LC and an isotropic liquid under an external electric field [31]. Figure 10 shows the schematic design, in which the nematic LC and the isotropic liquid are used for the main stream and the surrounding sub-streams, respectively. When an external electric field is applied in the direction perpendicular to the substrate, the LC molecules are reoriented along the electric field, resulting in the birefringent effect in the liquid layer. As *n_e_* and *n_o_* have different values in the nematic LC, the RI difference (i.e., the RI contrast on two sides of the interface) is dependent on the polarization state of the incident light. For the p-polarized light, the effective index equals to *n_o_*, which is smaller than *n_e_*. Therefore, the RI contrast (Δ*n* = *n_eff_* − *n_i_*) of the p-mode is smaller than that of the s-mode. As a consequence, the s-mode has a smaller focal length (see Figure 10b) than that of the p-mode (Figure 10a). This new type of hydrodynamic optofluidic lens can be modulated by either the flow rate or the polarization of the incident light.

**Figure 11:**
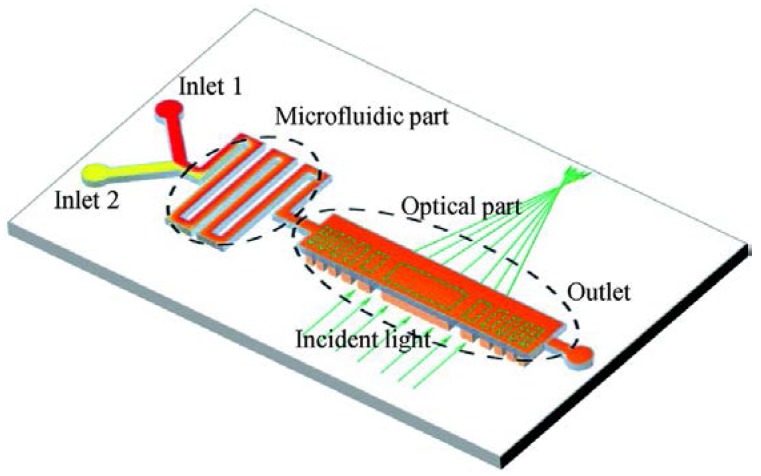
Schematic of the optofluidic Fresnel zone plate (FZP) [32]: it consists of a microfluidic part and an optical part. The former is used to prepare the mixed solution and the latter acts as the tunable FZP.

The traditional lenses focus the light due to the reflection or refraction. While in a Fresnel zone plate (FZP), the light is focused by diffraction, which is wavelength dependent. Inspired by this idea, Yang’s group demonstrated an optofluidic FZP used a solid-liquid hybrid structure [32] (see Figure 11). It utilizes a microfluidic mixer to prepare the liquid with a specific RI, which is delivered to fill the haft wave zone in the FZP. The radius *R_m_* of the *m*th half-wave zone is described by

(6) $$R_{m}=\sqrt{m\lambda f}$$

where *λ* and ƒ are the wavelength and the focal length, respectively. When *m* is an integer, a constructive interference appears at the focus. By tuning the RI of the liquid, it demonstrated the real-time modulation of the optical properties, such as peak intensity, spot size and focal length. In addition, this diffractive device is wavelength sensitive, which can selectively focus the desired wavelength. It also enables the switching between focusing, defocusing and collimation by the flow-rate control.

## 3. Applications of In-Plane Liquid Lenses

The in-plane optofluidic lenses can be used not only for beam manipulation, but also for some other applications in microfluidic networks, such as particle trapping. Optical tweezers utilize the tightly focused beam to trap particles in the microscale size, providing a nondestructive way to manipulate microparticles. The beam is usually focused by an objective lens with large NA and short focal length. In the traditional optical tweezer, the optical alignment is complicated, making it difficult to integrate it into a miniature lab-on-a-chip system. The emergence of optofluidics makes it easy to manipulate the particle using optical tweezers. In addition, sorting and precise moving of particle in 2D area can be easy achieved in optofluidic networks, which are difficult in conventional optical tweezers.

**Figure 12:**
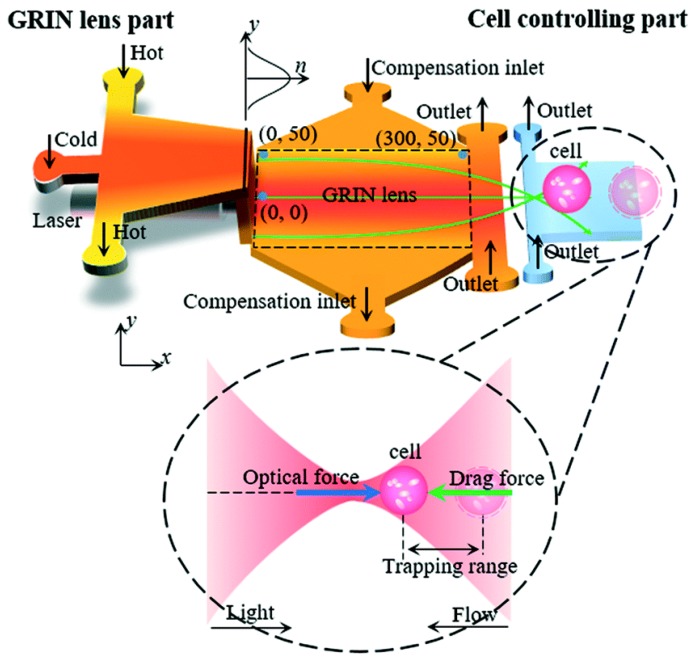
The schematic design of fluidic thermal GRIN lens for cell manipulation [30]: The system includes a lens chamber and a cell trapping chamber. Five streams at different temperatures are injected into the microfluidic chip to form a gradient refractive index across the channel.

Yang’s group proposed a reconfigurable optofluidic thermal GRIN lens and used it for single cell manipulation in the optofluidic chip [30]. As shown Figure 12, the system consists of two parts: the GRIN lens part and the cell trapping chamber. In the GRIN lens section, benzyl alcohol streams at different temperatures are pumped into the chamber to form a GRIN lens to focus the probe beam. And the living cell is contained in the cell trapping part. The cell is trapped and moved by the focused beam. By regulating the thermal lens, the trapping of the living cell can be modulated at a range of 280 µm. Recently, Aiqun Liu’s group has demonstrated the manipulation of particles with sizes of several 10s nanometers using optical potential wells created by an in-plane focused beam and fluidic constraints in optofluidic chips [47,48]. The combination of the quasi-Bessel optical profile and the loosely overdamped potential wells enable the precise manipulation of nanoparticles in the optofluidic channel. They revealed an unprecedentedly meaningful damping scenario that enriches our fundamental understanding of particle kinetics in intriguing optical systems and offered new opportunities for tumor targeting, intracellular imaging, and sorting small particles such as viruses and DNA [48]. The development in particle manipulation using the in-plane beams predicts perspective of the in-plane liquid lens.

**Figure 13:**
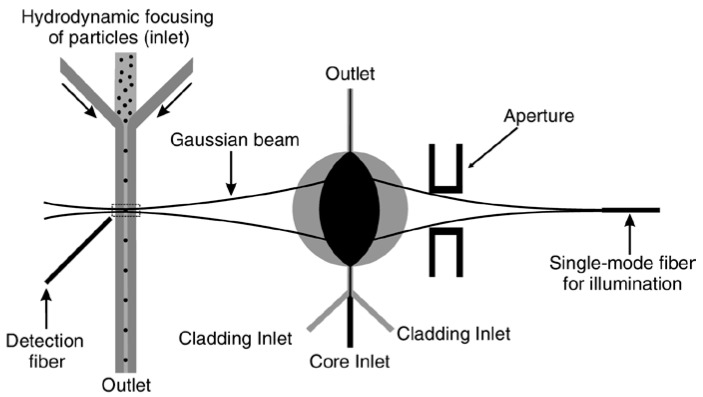
Schematic design of a flow cytometer using optofluidic lens [52].

The in-plane liquid lens has also been used for particle detection. Flow cytometers have been widely used for particle analysis, sorting and counting. A conventional flow cytometer consists of four main parts, including flow control, light guiding, signal collection and subsequent processing. By the use of optofluidic techniques, the flow cytometer can be integrated into a microfluidic system, reducing the size of the system and making it more portable and durable. The integration allows the benefits of including new optical features on the devices, such as built-in optical alignment, beam shaping, high optical sensitivity and high accuracy [49]. Zhang et al. gave a comprehensive review about the development of optofluidics based flow cytometers [49]. As the detected particles (or cells) are focused to a narrow stream to ensure that they pass through the optical interrogation point one by one, the measured coefficient of variation (CV) of fluorescent beads is strongly dependent on the beam geometry and bead size. As the beam coming out of the waveguide becomes divergent while it strikes at the interrogation region, a 2D lens is used to reshape and focus the beam, enhancing the beam quality for interrogation. In the past, a micro solid lens was fabricated in between the waveguide and the microchannel to improve the performance of the flow cytometer [50]. The precise fabrication of the waveguide and the solid lens system is required to ensure a good focused spot. While a reconfigurable in-plane lens allows the flexible modulation of the beam after fabrication. In addition, the tuning of the spot size makes it possible to get a low CV while detecting particles with different sizes. Goldin et al. proposed an in-plane liquid-filled lens for flow cytometer [51]. Figure 13 shows an optofluidic flow cytometer by Nguyen’s group [52]. It utilized a liquid core/liquid cladding lens to focus the light into a microchannel, where the detected particles flow through. A detection fiber was placed at the opposite side of the sample channel to collect the optical signal for subsequent processing. This compact device incorporated the optical elements and flow control part into a single chip to form a flow cytometer, instead of using bulky optics. This optofluidic flow cytometer demonstrated high efficiency and accuracy on particle counting and sizing. The detection of particles with sizes of 5, 10 and 20 µm was demonstrated. A better focused beam can significantly enhance the performance of the flow cytometers. Recently, Yang et al. gave a detail review on the development of the micro-optics for microfluidic analytical applications [53].

## 4. Discussion

Different types of in-plane optofluidic lenses have been demonstrated, providing numerous methods to manipulate the light in a microfluidic network. In the previous refractive lenses, the liquid-liquid (liquid-air) interfaces are spherical, which may lead to longitudinal spherical aberration (the marginal rays are focused closer to the lens than the paraxial rays). Therefore, the beam can not be well focused into the size close to the diffraction limit. The large focused spot size limits the practical applications. For instance, the CVs in the liquid-lens-based (beam waist 23 µm) cytometer [52] are not as good as those conventional cytometers. Howver, the aberration can be well-suppressed in the fluidic GRIN lenses [29,30,44]. For example, the well-designed thermal lens (the spot size 4 µm) has been used to manipulate the living cells in a microfluidic network [30]. As the reflective liquid lens is modulated through pressure control or hydrodynamics, it promises better robustness. While the liquid GRIN lenses usually use the solution diffusion or the temperature gradient, which are susceptible to the ambient fluctuation. In terms of response time, the interfacial deformation takes a few seconds to switch to a new balance state. In comparison, a thermal-induced liquid lens has a faster modulated speed (it is 200 ms in [29]). Another limitation is that most of previous in-plane liquid lenses require continuous supply of liquids, which consumes a lot of liquid and reduces the compatibility of the devices. Although there are still some drawbacks, the in-plane optofluidic lenses find useful applications in lab-on-a-chip systems. In addition, the LC-based and FZP-based liquid lenses enable the polarization and wavelength manipulation in a microfluidic chip. It is foreseeable that more liquid lenses will be incorporated into a chip to achieve versatile microsystems.

## 5. Conclusions

This article presents a general review of in-plane optofluidic lenses. Based on their working principles and operation methods, they are categorized into three types: refractive lens, gradient index lens and others. The first two cover most of the reported designs, and the last represents some new types, such as birefringent optofluidic lenses and Fresnel zone plate liquid lenses. The in-plane optofluidic lenses have wide tunability, provide a flexible way to manipulate the beam in microfluidic networks and can be easily integrated with lab-on-a-chip systems, making them suitable for transportation and detection of the particle at the same time. It is foreseeable that the in-plane liquid lenses will make the optofluidic networks a versatile platform for research and practical applications. For instance, combing the in-plane beam shaping and microfluidic techniques enables the manipulation and analysis of the particle or cells down to nanometer scale, which may broaden our fundamental understanding of particle kinetics and be useful for biological research. A tightly focused in-plane beam can be used for sample (particle/cell) traping, transportation, separation and detection, making it possible to construct a portable integrated system for biochemical applications.
